# Supplementary material for: Design, Synthesis, and Biological Evaluation of N-Acylhydrazones and Their Activity Against Leishmania amazonensis Promastigotes
Source: Microorganisms. 2025 Jul 2;13(7):1563. doi: 10.3390/microorganisms13071563 (PMC12298860; doi:10.3390/microorganisms13071563)
Supplement: Supplementary file 1 [file microorganisms-13-01563-s001.zip › RMN amostras-20241114T155656Z-001.zip!/RMN amostras/composto 28 - PMM 27-18 13C.pdf]

PMM 27-18 – Joao Mafra
Op. Eliane

farmanguinhos
Instituto de Tecnologia em Fármacos

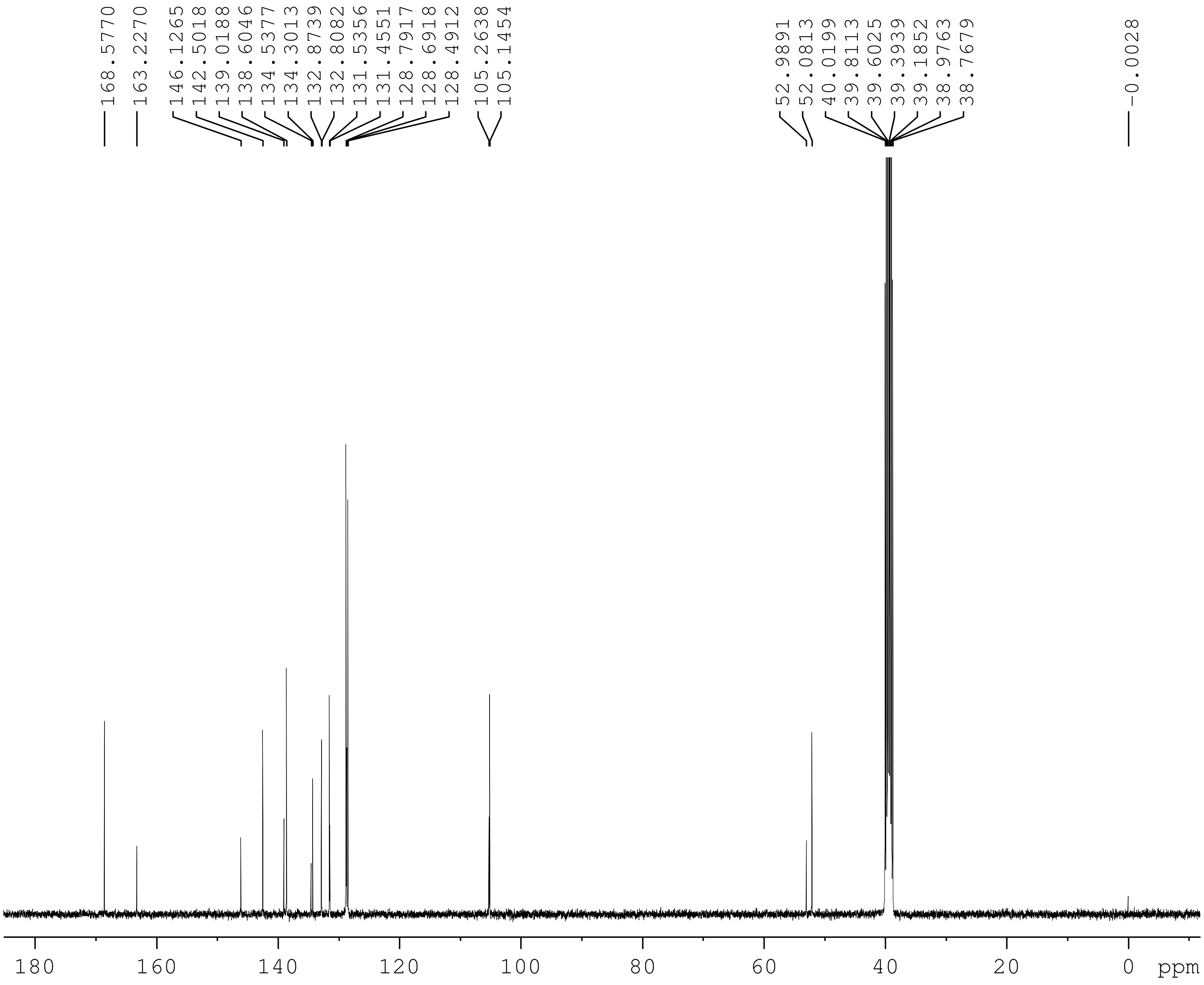


Current Data Parameters

| | |
|---|---|
| NAME | si107182332 |
| EXPNO | 11 |
| PROCNO | 1 |

F2 - Acquisition Parameters

| | |
|---|---|
| Date_ | 20180725 |
| Time | 12.40 h |
| INSTRUM | spect |
| PROBHD | Z122623_0041 ( |
| PULPROG | zgpg30 |
| TD | 65356 |
| SOLVENT | DMSO |
| NS | 2048 |
| DS | 4 |
| SWH | 24038.461 Hz |
| FIDRES | 0.735616 Hz |
| AQ | 1.3594048 sec |
| RG | 53.19 |
| DW | 20.800 usec |
| DE | 18.00 usec |
| TE | 298.0 K |
| D1 | 1.00000000 sec |
| D11 | 0.03000000 sec |
| TD0 | 1 |
| SFO1 | 100.6278593 MHz |
| NUC1 | 13C |
| P1 | 10.00 usec |
| PLW1 | 35.50000000 W |
| SFO2 | 400.1516006 MHz |
| NUC2 | 1H |
| CPDPRG[2 | waltz16 |
| PCPD2 | 90.00 usec |
| PLW2 | 7.30000019 W |
| PLW12 | 0.11919000 W |
| PLW13 | 0.05995100 W |

F2 - Processing parameters

| | |
|---|---|
| SI | 32768 |
| SF | 100.6178559 MHz |
| WDW | EM |
| SSB | 0 |
| LB | 1.00 Hz |
| GB | 0 |
| PC | 1.40 |
